# Supplementary material for: Evolution of thiolate-stabilized Ag nanoclusters from Ag-thiolate cluster intermediates
Source: Nat Commun. 2018 Jun 18;9:2379. doi: 10.1038/s41467-018-04837-x (PMC6006296; doi:10.1038/s41467-018-04837-x)
Supplement: Supplementary file 1 — Supplementary Information [file 41467_2018_4837_MOESM1_ESM.pdf]

Supplementary Information

Evolution of thiolate-stabilized Ag nanoclusters from Ag-thiolate cluster intermediates

Cao et al.

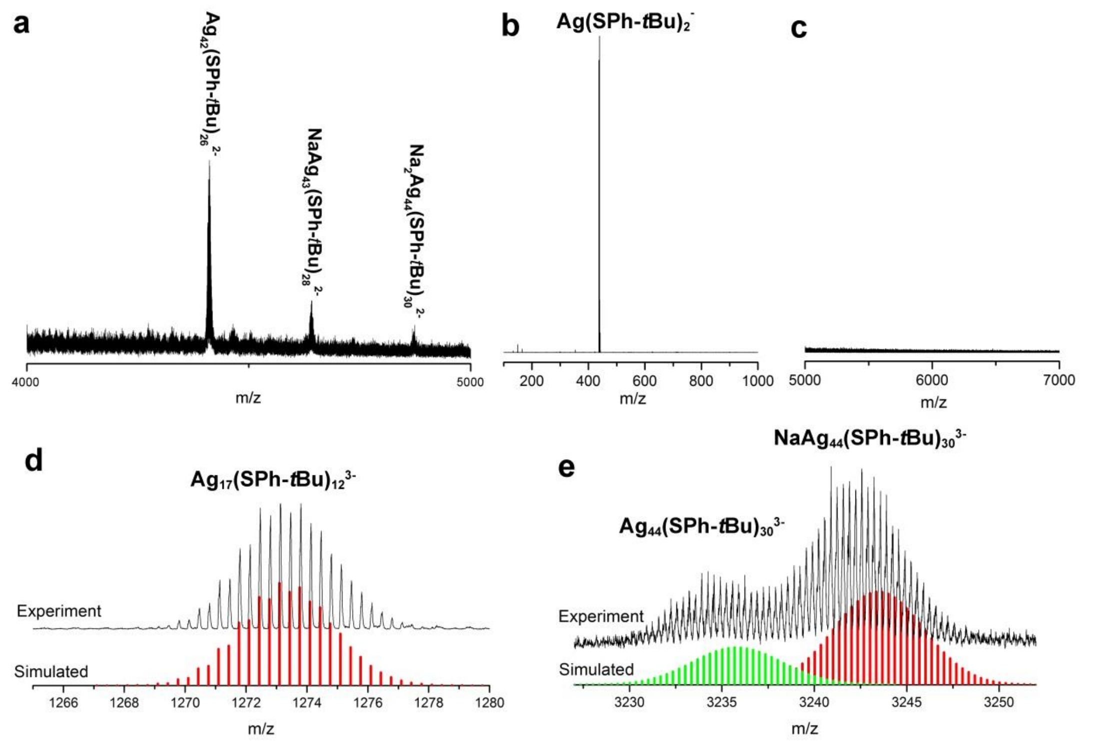

Supplementary Figure 1 | Analysis of ESI-MS spectra of the final products. (a) Assignment of peaks between *m/z* 4000-5000 in the ESI-MS spectrum of the final product obtained using SPh-*t*Bu as the starting thiol. (b) ESI-MS spectrum in the range of *m/z* 100-1000. Ag(SPh-*t*Bu)$^-$ was detected as the main fragment. (c) ESI-MS spectrum in the range of *m/z* 5000-7000. No signals for large species were detected. (d) Comparison of the experimental and simulated mass spectra for $Ag_{17}(SPh\text{-}tBu)_{12}^{3-}$. (e) Comparison of the experimental and simulated mass spectra for $Ag_{44}(SPh\text{-}tBu)_{30}^{3-}$ and $NaAg_{44}(SPh\text{-}tBu)_{30}^{3-}$. The ESI-MS patterns of Ag_{44} and Ag_{17} were similar to those reported in previous works[1-3].

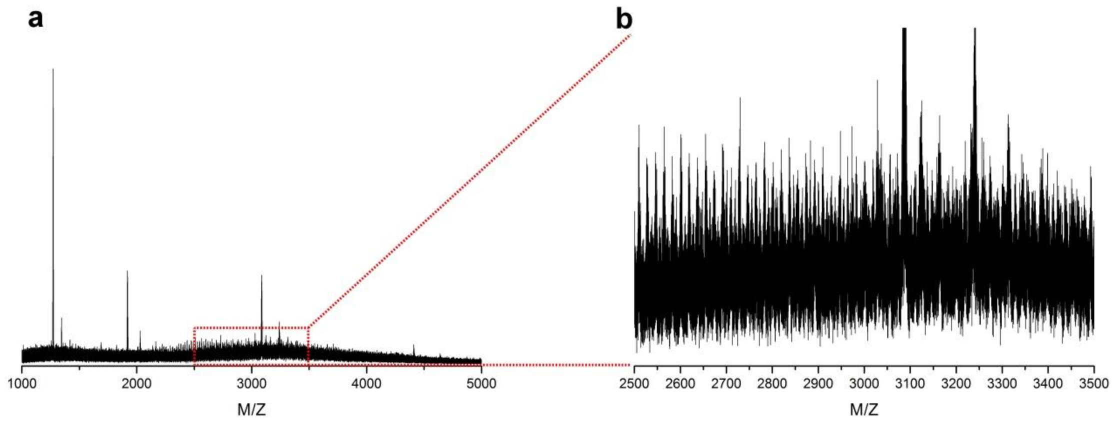

Supplementary Figure 2 | Intermediate signals for the original reaction conditions. (a) ESI-MS spectrum captured during a typical reaction with SPh-*t*Bu as the starting thiol and (b) expanded view of the spectrum in the range of *m/z* 2500-3500. Weak peaks of the intermediates were observed.

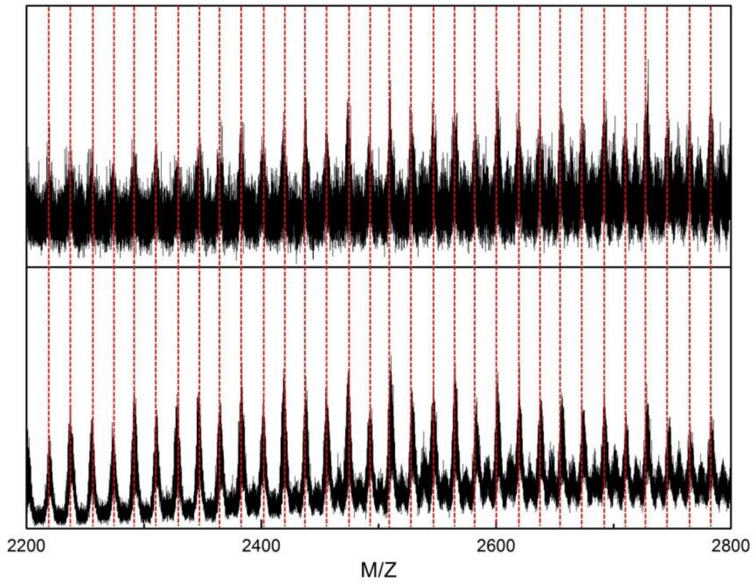

Supplementary Figure 3 | Comparison of the intermediate species captured in two different conditions. The intermediate species without NaOH (top panel) have perfect coincidence with the intermediate species shown in Fig. 3a (bottom panel) in the range of *m/z* 2200-2800. Evidence is found for 32 species.

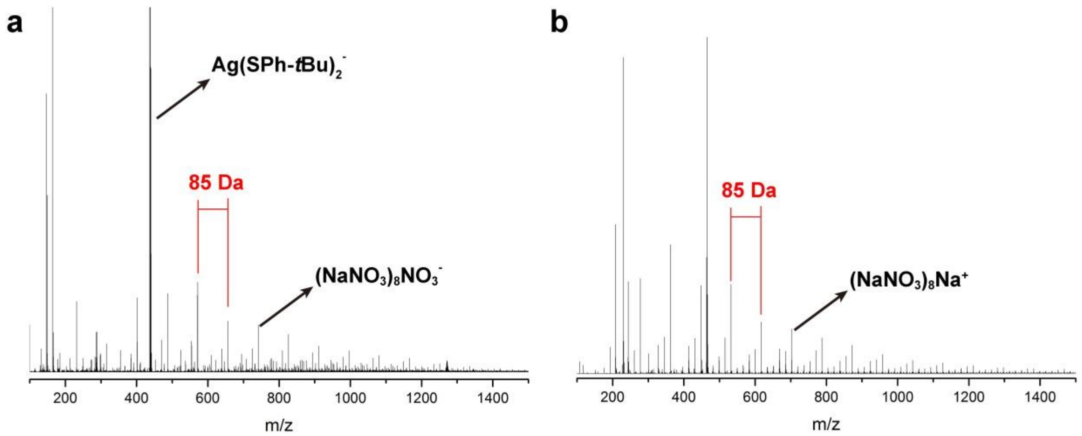

Supplementary Figure 4 | Assignment of ESI-MS peaks in the low molecular weight region. (a) Negative-mode and (b) positive-mode ESI-MS spectra of the intermediate reaction mixture in the range of *m/z* 100-1500. A series of peaks without Ag isotopic patterns were observed and they were likely associated with inorganic ions such as Na^+ and NO_3^- in the reaction mixture. As shown in the figures, these peaks were regularly spaced by 85 Da corresponding to the molecular weight of $NaNO_3$. The main peaks are therefore assigned to species containing multiple $NaNO_3$ and an extra Na^+ or NO_3^- to give positive or negative charge, respectively. The intensities of the peaks associated with the $(NaNO_3)_xNO_3^-$ and $(NaNO_3)_xNa^+$ ions decreased during the reaction process (Fig. 2).

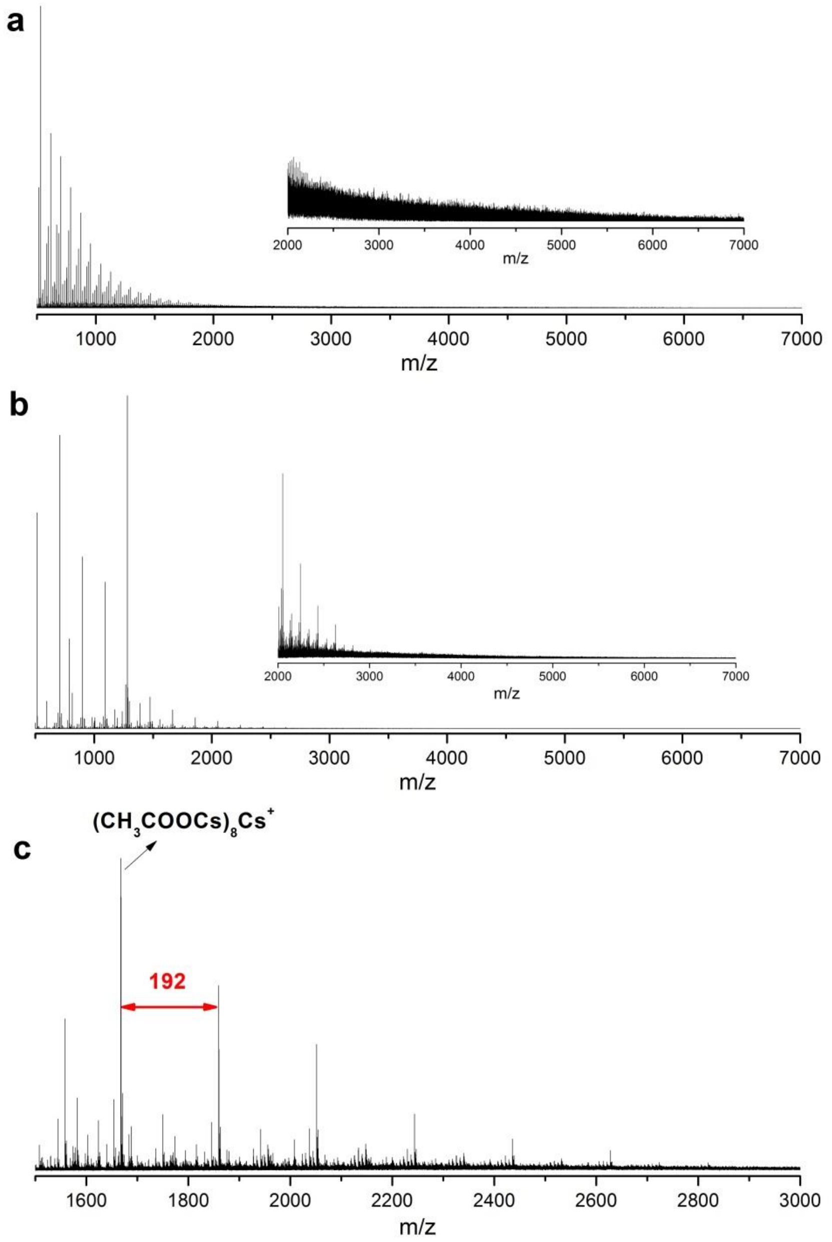

Supplementary Figure 5 | Detection of positively charged and neutral intermediates in the reaction mixture. (a) Positive-mode ESI-MS spectrum of the intermediate reaction mixture in the range of *m/z* 500-7000. The spectrum shows a series of peaks associated with inorganic ions such as Na^+ and NO_3^- in the reaction mixture (Supplementary Fig. 4b). The inset shows an expanded view of spectrum in the range of *m/z* 2000-7000. No obvious signals were observed. (b) Positive-mode ESI-MS spectrum of the intermediate reaction mixture in the range of *m/z* 500-7000. 50 mM CH_3COOCs was added to facilitate ionization of neutral species[4-7]. Inset shows an expanded view of spectrum in the range of *m/z* 2000-7000. Except for a series of species related to Cs^+ and CH_3COO^- in the low molecular weight region, no intermediate species could be detected in the range of *m/z* 2000-7000. (c) Expanded view of spectrum obtained after the addition of CH_3COOCs in the range of *m/z* 1500-3000. Series of peaks were regularly spaced by 192 Da, corresponding to the molecular weight of CH_3COOCs. The main peaks could be assigned to species with multiple CH_3COOCs and an extra Cs^+ to give positive charge.

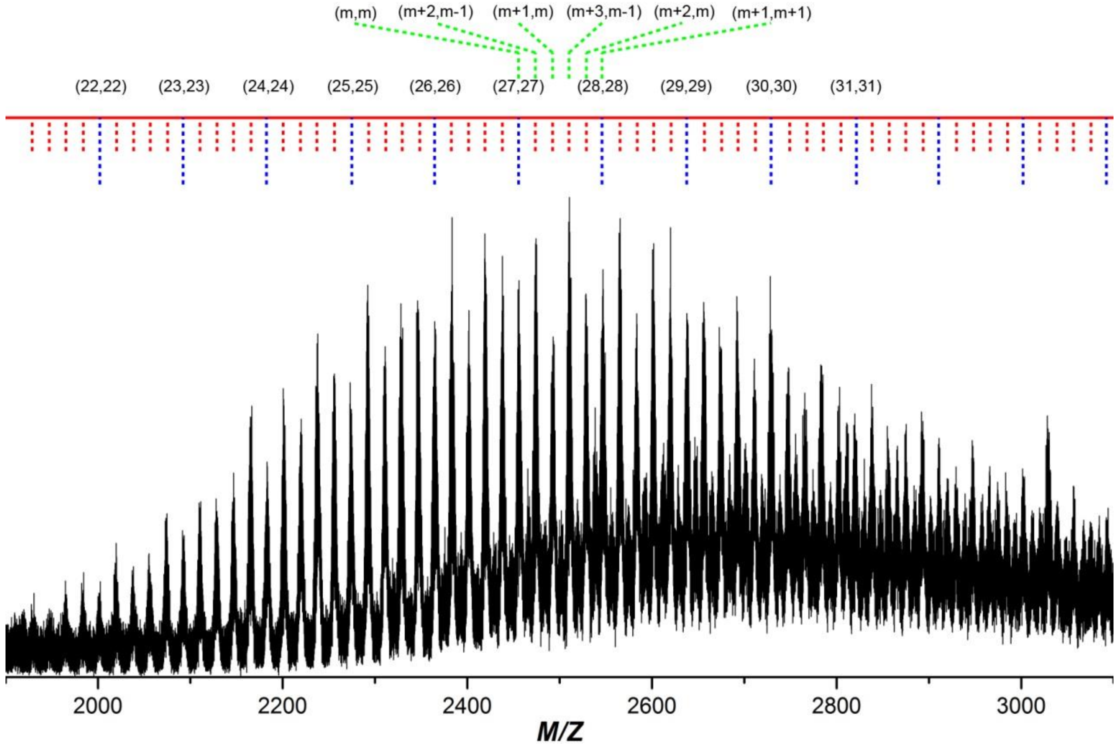

Supplementary Figure 6 | Assignment of peaks associated with the intermediate species of $Ag_{17}(SPh\text{-}tBu)_{12}^{3-}$. All peaks can be assigned to Ag-thiolate clusters with a -3 charge: The notation (*a*, *b*) is used, where *a* is the number of Ag atoms and *b* is the number of thiolate ligands in the clusters, respectively. *m* refers to the number of both Ag atoms and thiolate ligands in Ag-thiolate clusters with a formula of $Ag_m(SPh\text{-}tBu)_m^{3-}$. The peaks between (*m*, *m*) and (*m*+1, *m*+1) were assigned to (*m*+2, *m*-1), (*m*+1, *m*), (*m*+3, *m*-1) and (*m*+2, *m*), respectively (See Supplementary Fig. 7 below for more detailed assignments). In our analysis, formulas with *a*-*b*>4 or *a*-*b*<0 were not considered, because in thiolate-stabilized Ag_{17} nanoclusters *a*-*b*=5 and in the Ag-thiolate complex precursor *a*-*b*=0.

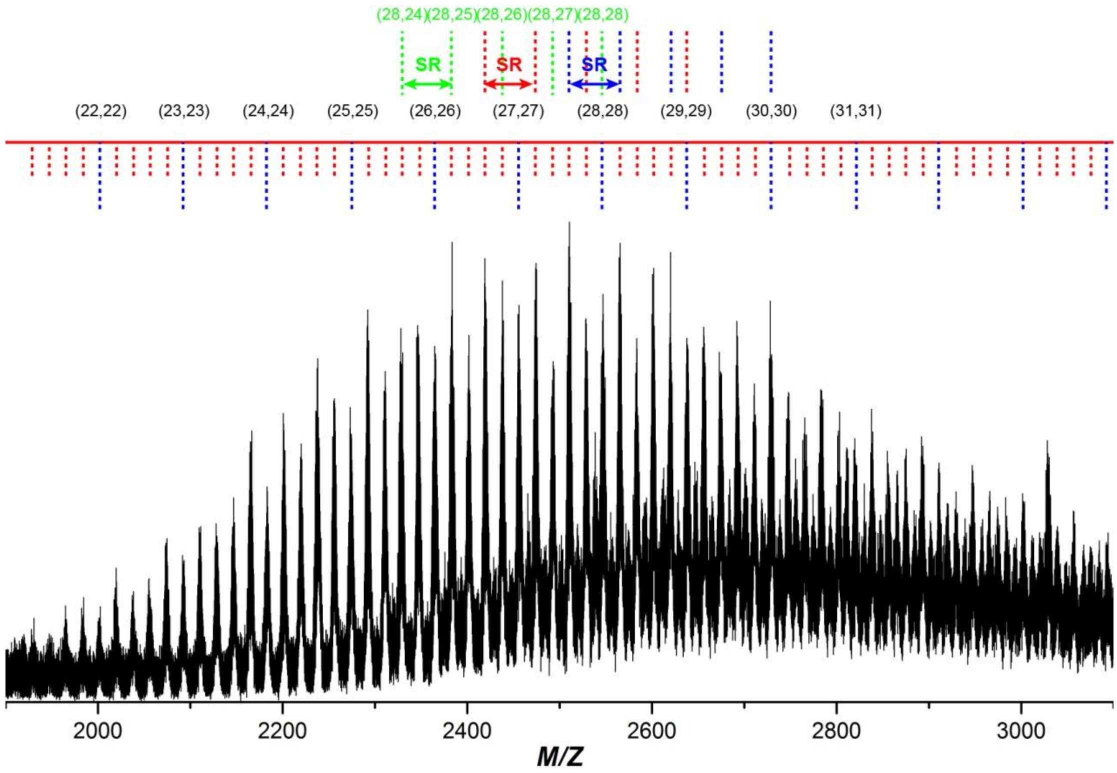

Supplementary Figure 7 | Assignment of peaks for the intermediate species of $Ag_{17}(SPh\text{-}tBu)_{12}^{3-}$ labeled by red dashed lines. Green dashed lines label a series of peaks with formulas of (28, 24), (28, 25), (28, 26), (28, 27) and (28, 28), respectively. Each peak differs by one thiolate ligand to the adjacent peak. By searching for similar series of peaks, we were able to assign all the peaks in the ESI-MS spectrum. As shown in the figure, peaks between (27, 27) and (28, 28) could be successfully assigned to (29, 26), (28, 27), (30, 26) and (29, 27).

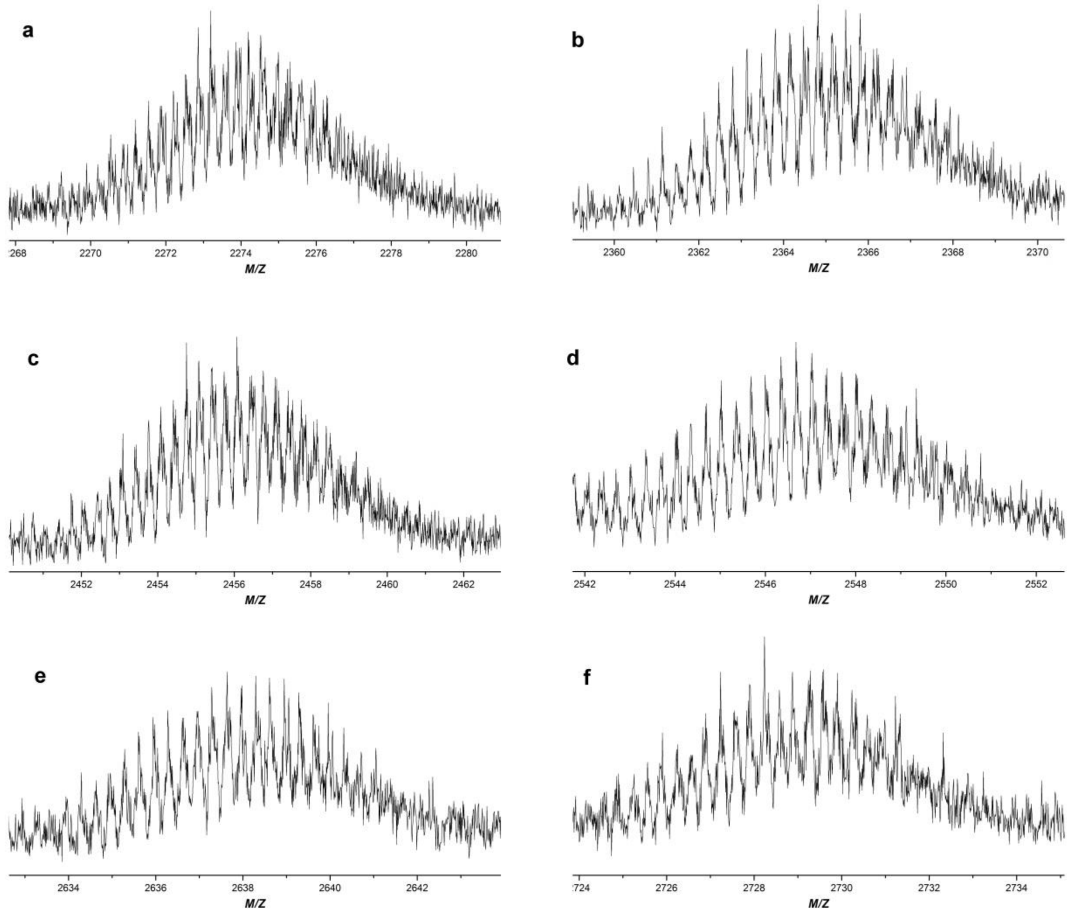

Supplementary Figure 8 | Expanded view of the ESI-MS spectra for selected intermediate species of $Ag_{17}(SPh\text{-}t\text{Bu})_{12}^{3-}$. All peaks can be assigned to Ag-thiolate clusters with a -3 charge. Ag isotopic patterns can be observed. (a) peak (25, 25), (b) peak (26, 26), (c) peak (27, 27), (d) peak (28, 28), (e) peak (29, 29) and (f) peak (30, 30). For more details about each peak, see Supplementary Table 1. The relationship of these intermediate species with the formation of Ag_{17} was explored in Supplementary Fig. 9 below.

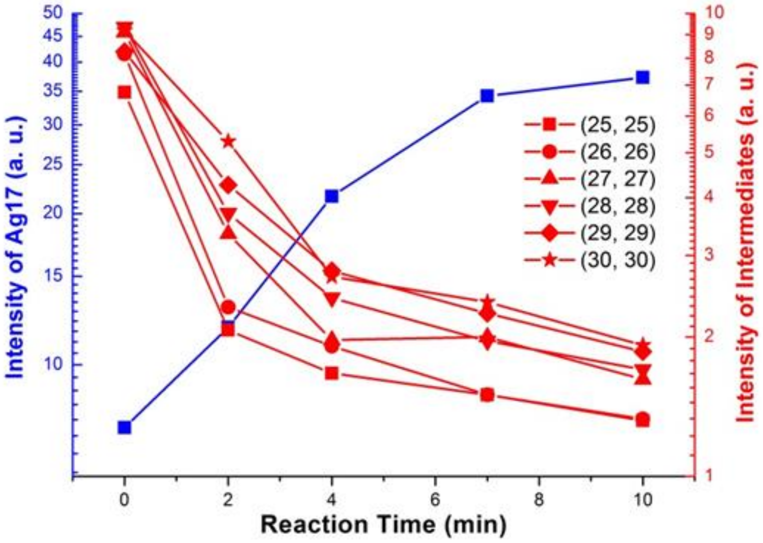

Supplementary Figure 9 | Variation of the intensities of intermediate species in the formation process of $Ag_{17}(SPh\text{-}tBu)_{12}^{3-}$. Intensity of each species was calculated by the signal-to-noise ratio of the maximum of each series of peaks. Calculation of the intensity of Ag_{17} is based on the peak corresponding to $Ag_{17}(SPh\text{-}tBu)_{12}^{3-}$. Note that the peak is nearly invisible when the signal-to-noise ratio is less than 2. The disappearance of the various intermediates coincides with the appearance of Ag_{17}.

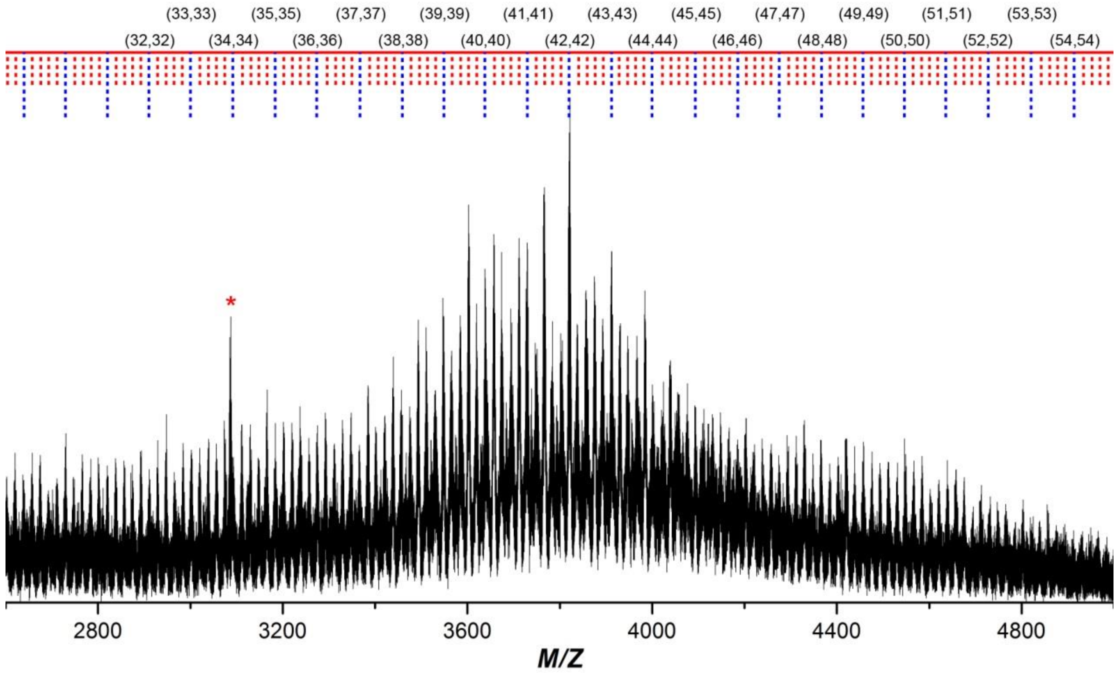

Supplementary Figure 10 | Assignment of peaks associated with the intermediate species of $Ag_{44}(SPh\text{-}tBu)_{30}^{4-}$ in Fig. 3d. Peaks labeled by dashed red lines were assigned in the same way as described in Supplementary Fig. 6. The peak marked by an asterisk is the main fragment of $Ag_{44}(SPh\text{-}tBu)_{30}^{4-}$ in ESI-MS (i.e., $Ag_{43}(SPh\text{-}tBu)_{28}^{3-}$).

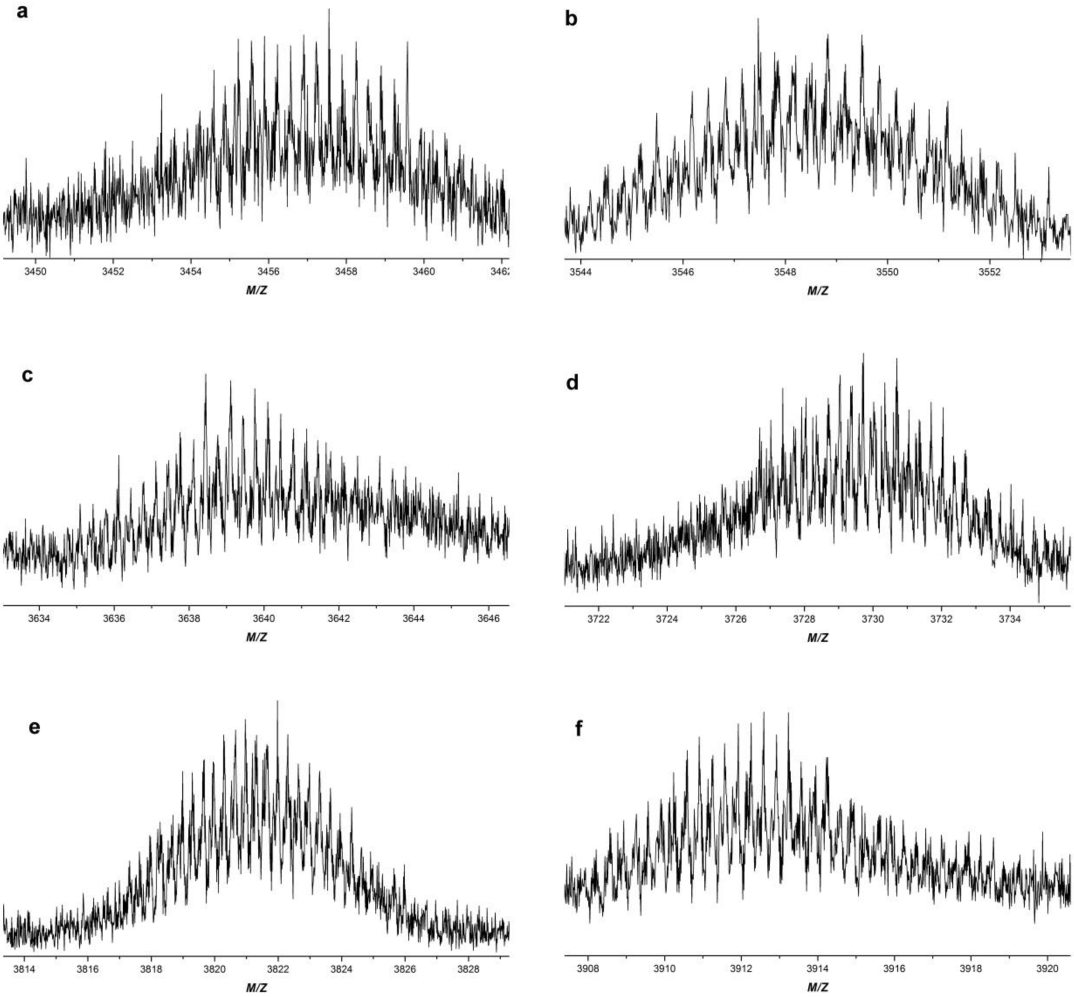

Supplementary Figure 11 | Expanded view of the ESI-MS spectra for selected intermediate species of $Ag_{44}(SPh\text{-}tBu)_{30}^{4-}$. All peaks can be assigned to Ag-thiolate clusters with a -3 charge. Ag isotopic patterns can be observed. (a) Peak (38, 38). (b) Peak (39, 39). (c) Peak (40, 40). (d) Peak (41, 41). (e) Peak (42, 42). (f) (43, 43). See Supplementary Table 2 for further details about the peaks.

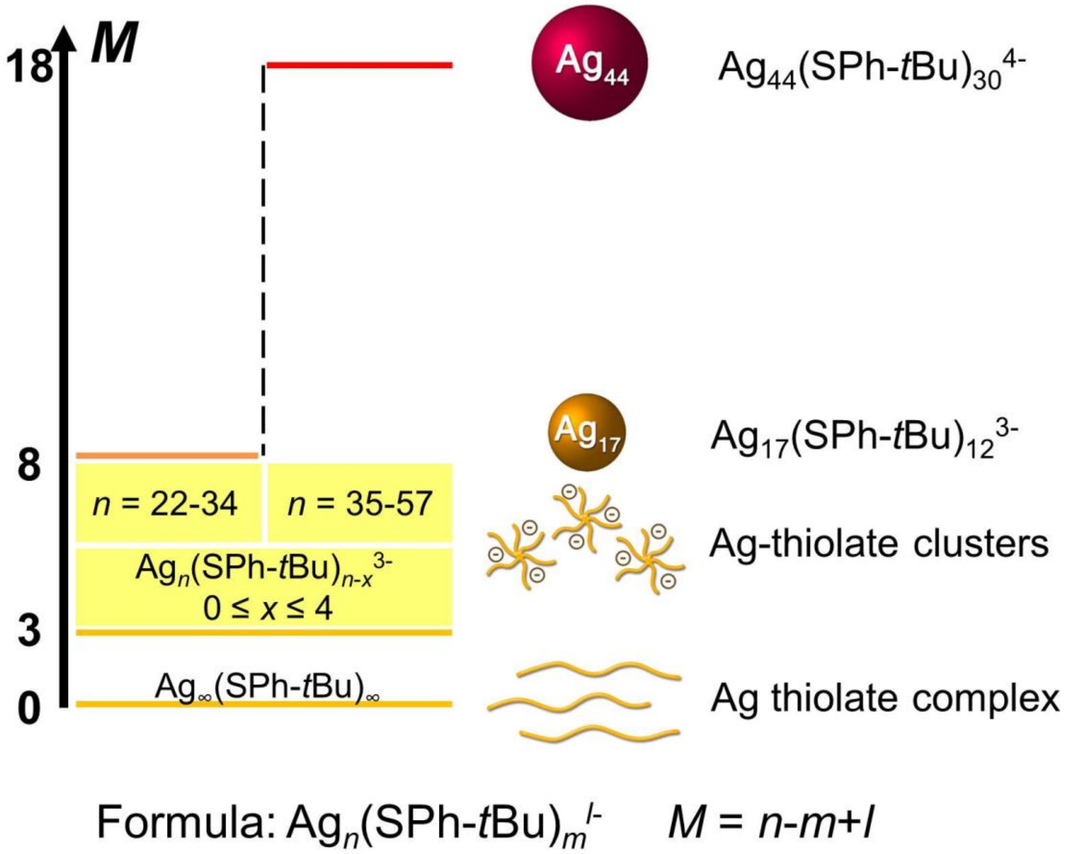

Supplementary Figure 12 | Schematic illustration of the evolution of *M* during the chemical reduction of the Ag-thiolate complex precursor to thiolate-stabilized Ag nanoclusters.

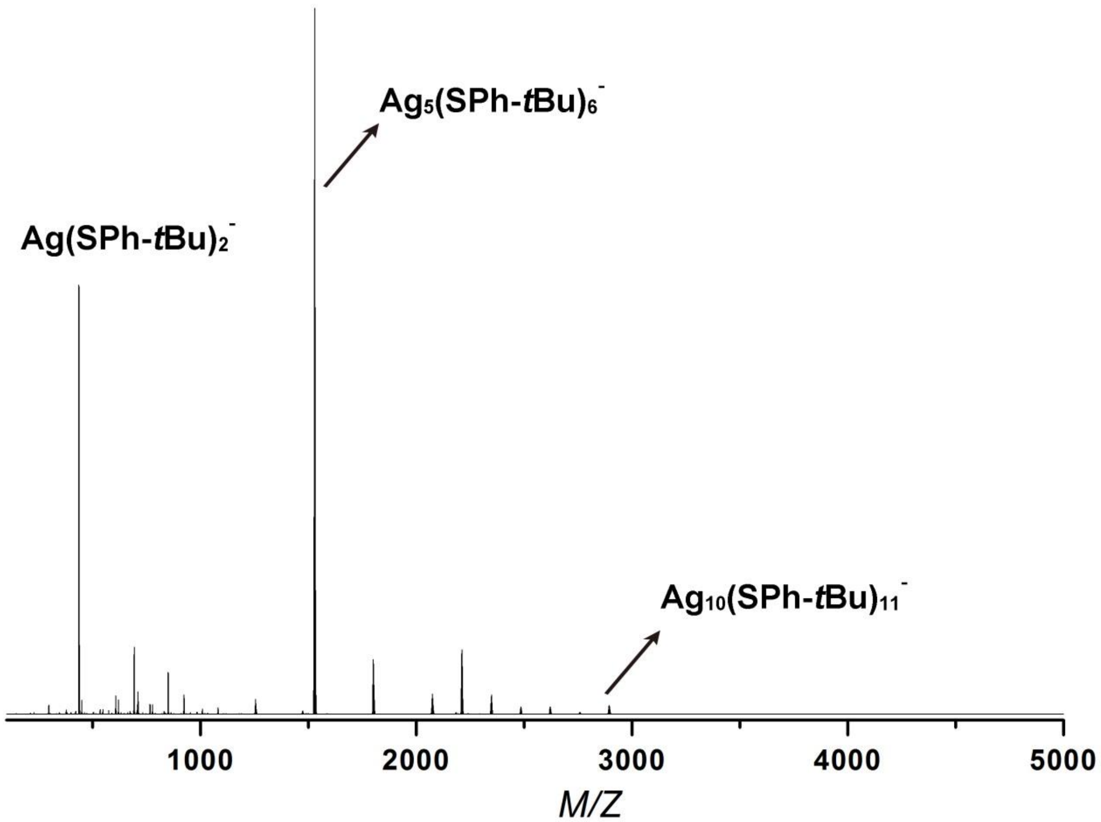

Supplementary Figure 13 | ESI-MS spectrum of the colorless solution obtained after the final $Ag_{17}(SPh\text{-}tBu)_{12}^{3-}$ and $Ag_{44}(SPh\text{-}tBu)_{30}^{4-}$ products were etched. Only Ag-thiolate monomers and oligomers can be observed.

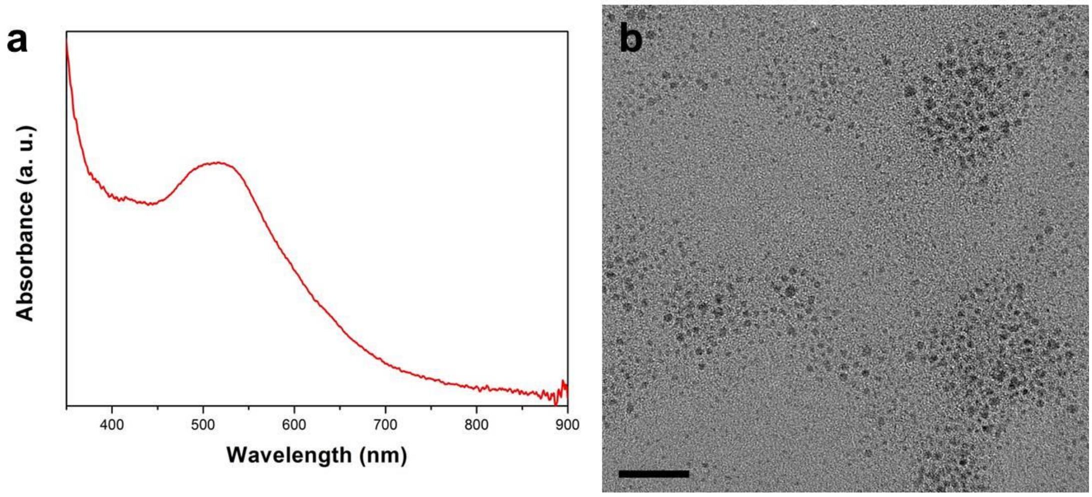

Supplementary Figure 14 | Characterization of the product obtained through a bottom-up process. (a) UV-vis absorption spectrum and (b) TEM image of the final product obtained through a bottom-up process. Scale bar: 20 nm. Ag nanospheres larger than 3 nm could be observed.

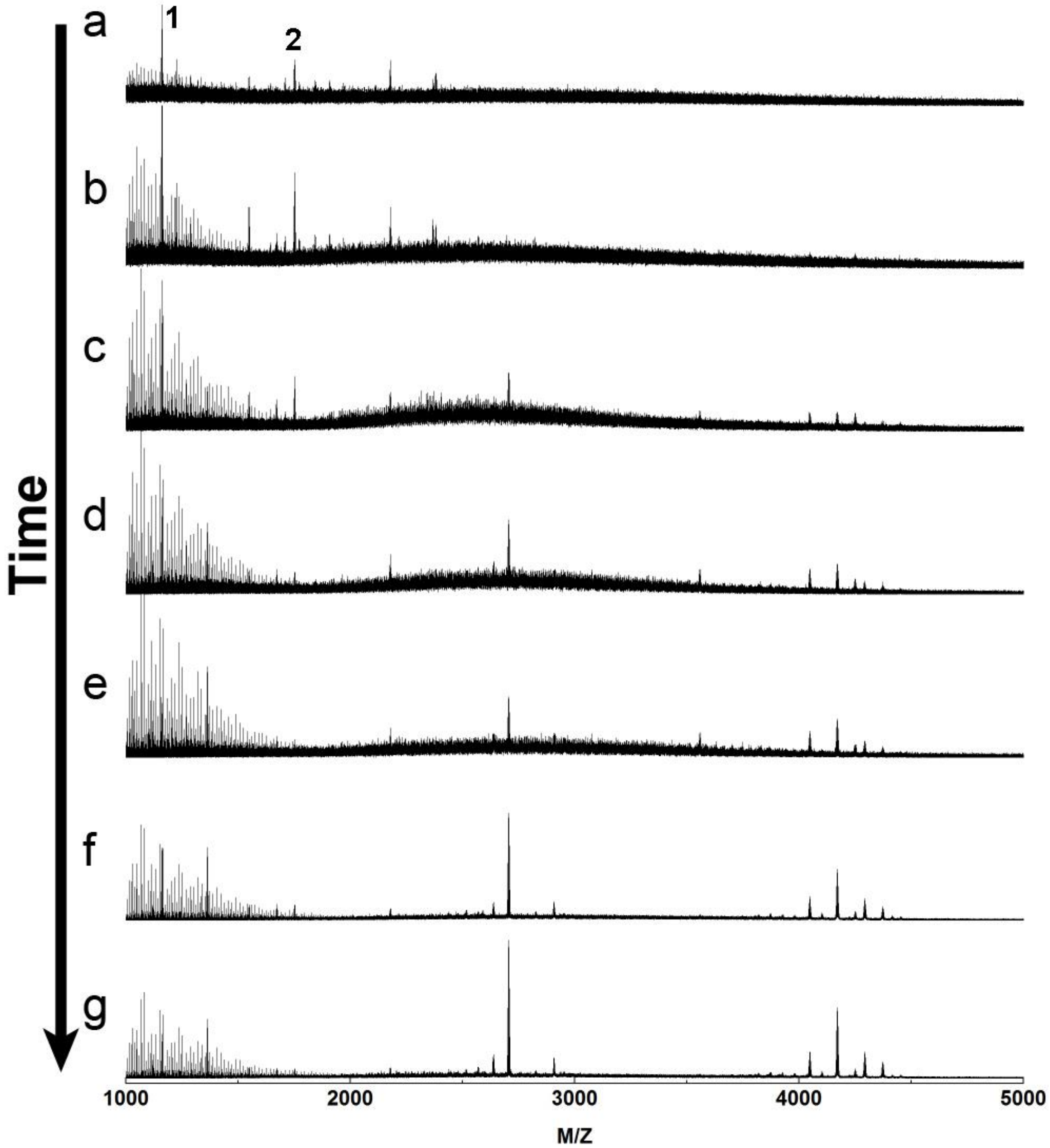

Supplementary Figure 15 | Time-dependent ESI-MS spectra for the evolution of thiolate-stabilized Ag nanoclusters using $HSPhMe_2$ as the starting thiol. An expanded view of the spectrum shown in (c) is provided in Supplementary Fig. 16. Peaks labeled 1 and 2 in (a) were thiolate-stabilized Ag_{17} nanoclusters, and their detailed analysis is shown in Supplementary Fig. 18. Reaction time: (a) 2 min, (b) 4 min, (c) 6 min, (d) 8 min, (e) 10 min, (f) 15 min, (g) 20 min.

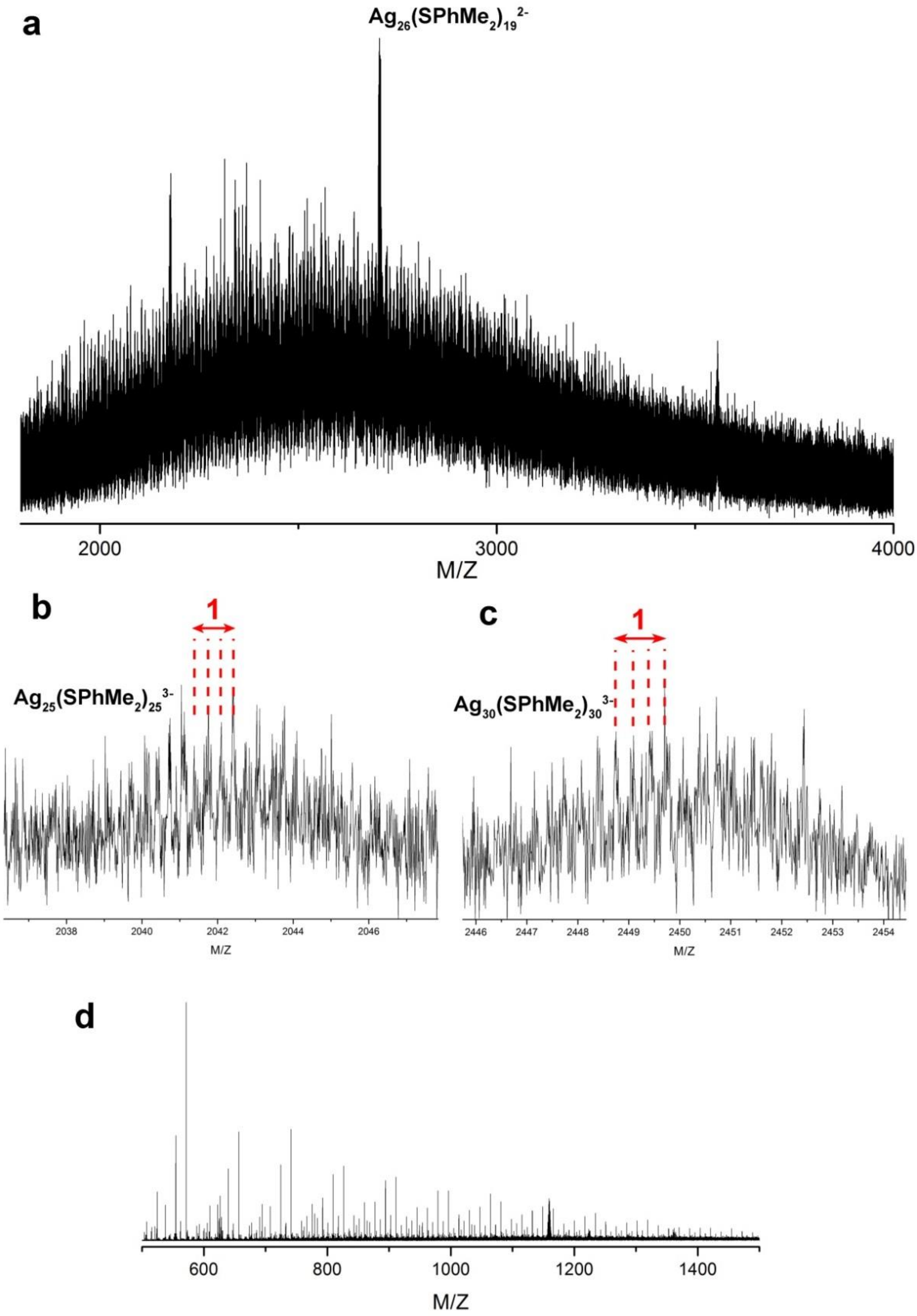

Supplementary Figure 16 | Detailed analysis of the species in Supplementary Fig. 15c. (a) Expanded view of the ESI-MS spectra of Supplementary Fig. 15c showing a series of intermediate species. Spectra (b) and (c) show the peaks typical for $Ag_{25}(SPhMe_2)_{25}^{3-}$ and $Ag_{30}(SPhMe_2)_{30}^{3-}$, respectively. All peaks are associated with the intermediate Ag-thiolate clusters with a -3 charge. (d) Expanded view of the ESI-MS spectra of Supplementary Fig. 15c in the range of *m*/*z* 500-1500. Except the peaks of Ag_{17} species, all the peaks have no characteristic isotropic peaks of Ag and can be assigned to salts, such as $NaBH_4$, in the reaction mixture.

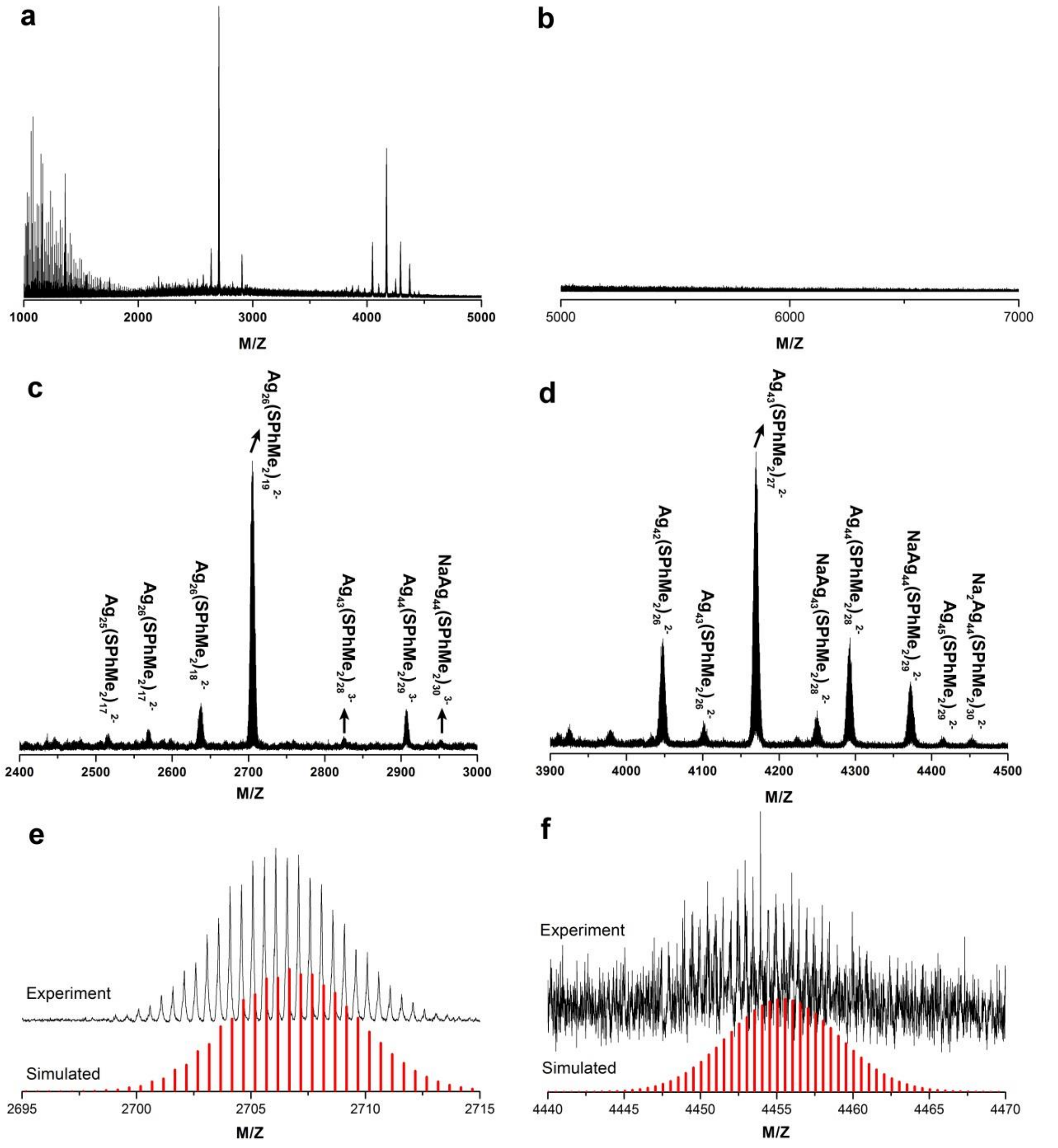

Supplementary Figure 17 | ESI-MS spectra of the final product obtained using $HSPhMe_2$ as the starting thiol. (a) ESI-MS spectrum in the range of *m/z* 1000-5000. Peaks in the range of *m/z* 1000-2000 belong to salts in the mixture. (b) ESI-MS spectrum in the range of *m/z* 5000-7000. No obvious signals can be observed, including Ag_{25} (at *m/z* ~5170)[8]. (c) and (d) show the assignments for the peaks. All peaks can be assigned to thiolate-stabilized Ag_{26}, Ag_{44} nanoclusters and their fragments. (e) Comparison of the experimental and simulated mass spectra of $Ag_{26}(SPhMe_2)_{19}^{2-}$. (f) Comparison of the experimental and simulated mass spectra of $Na_2Ag_{44}(SPhMe_2)_{30}^{2-}$. The fragment pattern of $Ag_{44}(SPhMe_2)_{30}^{4-}$ is different from that of $Ag_{44}(SPh\text{-}t\text{Bu})_{30}^{4-}$ under the same ESI-MS test conditions, indicating their different stabilities.

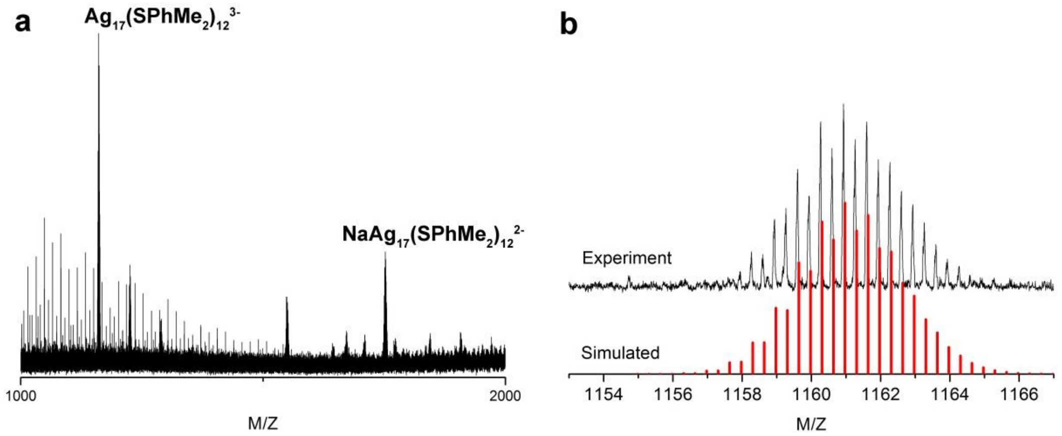

Supplementary Figure 18 | Analysis of the peaks labeled 1 and 2 in Supplementary Fig. 15a. (a) Peak assignments for thiolate-stabilized Ag_{17} nanoclusters formed during the reaction using $HSPhMe_2$ as the starting thiol. (b) Comparison of the experimental and simulated mass spectra of $Ag_{17}(SPhMe_2)_{12}^{3-}$.

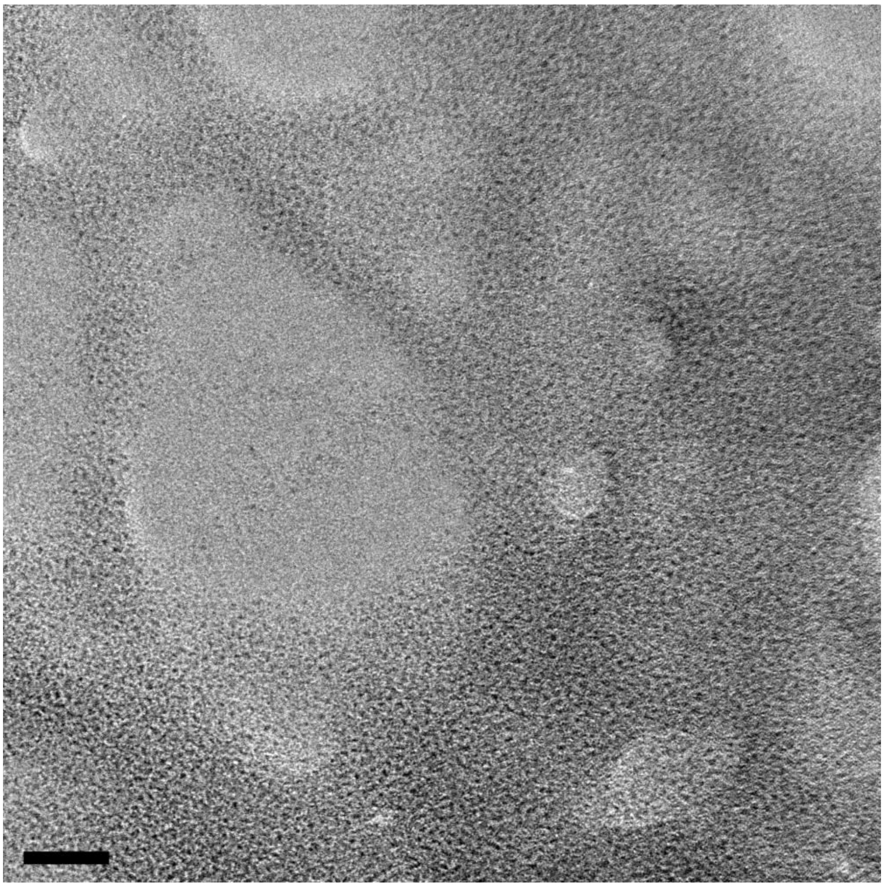

Supplementary Figure 19 | TEM image of the AgSPh-*t*Bu precursor. The yellow precipitate consists of aggregates of small nanoparticles with size of ~1-2 nm. Scale bar: 20 nm.

Supplementary Table 1 | ESI-MS peak positions for selected intermediate Ag-thiolate clusters of $Ag_{17}(SPh\text{-}tBu)_{12}^{3-}$ and their assignments.

Peak (*m/z*)[a]	Assigned Formula[b]	Theoretical Value (*m/z*)[c]	Error
2274.21	25,25	2276.22	2.02
2292.22	27,24	2293.04	0.82
2310.93	26,25	2312.18	1.25
2328.19	28,24	2329.00	0.81
2346.89	27,25	2348.14	1.25
2364.80	26,26	2367.27	2.47
2383.51	28,25	2384.09	0.58
2401.86	27,26	2403.23	1.37
2419.45	29,25	2420.05	0.60
2438.18	28,26	2439.18	1.00
2456.06	27,27	2458.32	2.26
2474.45	29,26	2475.14	0.69
2492.81	28,27	2494.28	1.47
2510.75	30,26	2511.10	0.35
2528.77	29,27	2530.23	1.46
2546.69	28,28	2549.37	2.68
2565.38	30,27	2566.19	0.81
2583.41	29,28	2585.33	1.92
2601.68	31,27	2602.15	0.47
2620.04	30,28	2621.28	1.24
2638.28	29,29	2640.42	2.14
2656.35	31,28	2657.24	0.89
2674.71	30,29	2676.38	1.67
2692.60	32,28	2693.19	0.59
2711.32	31,29	2712.33	1.01

a Centre position of the series of isotopic peaks.

b (*m*, *n*) stands for Ag_mSR_n, where SR is $C_{10}H_{13}S$. All the species carried a -3 charge.

c Calculation of the theoretical values was based on the average mass of Ag (107.8681 g mol^{-1}) and SR (165.2787 g mol^{-1}).

Supplementary Table 2 | ESI-MS peak positions for selected intermediate Ag-thiolate clusters of $Ag_{44}(SPh\text{-}tBu)_{30}^{4-}$ and their assignments[a].

Peak (*m/z*)	Assigned Formula	Theoretical Value (*m/z*)	Error
3087.12	43,28[b]	3088.71	1.59
3457.54	38,38	3459.86	2.32
3476.25	40,37	3476.68	0.43
3493.83	39,38	2495.82	1.99
3512.21	41,37	3512.63	0.42
3531.46	40,38	3531.77	0.31
3548.47	39,39	3550.91	2.44
3566.12	41,38	3567.73	1.61
3584.78	40,39	3586.86	2.08
3602.80	42,38	3603.68	0.88
3620.77	41,39	3622.82	2.05
3639.77	40,40	3641.96	2.19
3658.04	42,39	3658.78	0.74
3675.71	41,40	3677.91	2.20
3694.69	43,39	3694.73	0.04
3712.68	42,40	3713.87	1.19
3730.70	41,41	3733.00	2.30
3749.29	43,40	3749.83	0.54
3766.64	42,41	3768.96	2.32
3784.59	44,40	3785.78	1.19
3803.28	43,41	3804.91	1.63
3821.66	42,42	3834.06	2.40
3838.89	44,41	3840.87	1.98
3858.61	43,42	3860.01	1.40
3875.94	45,41	3876.83	0.89
3894.24	44,42	3895.97	1.73
3912.59	43,43	3915.10	2.71
3930.51	45,42	3931.92	1.41
3948.88	44,43	3951.06	2.18
3967.58	46,42	3967.88	0.30
3985.15	45,43	3987.02	1.87
4002.74	44,44	4006.15	3.31
4022.39	46,43	4022.97	0.60
4039.58	45,44	4042.11	2.53
4057.57	47,43	4058.93	1.36
4077.09	46,44	4078.07	0.98

a The analysis method was the same as that of Supplementary Table 1.

b (43,28) is the main fragment of Ag_{44} nanocluster in ESI-MS characterization.

Supplementary Note 1

Ag_{44} and Ag_{17} have relatively higher stability than other Ag nanoclusters. This can be partly rationalized by shell closing phenomenon, wherein nanoclusters containing 8, 18, 34, *etc.* with close shell electrons will have higher stability. The Ag_{17} and Ag_{44} nanoclusters contain 8 and 18 closed shell electrons, respectively. This explains why they are more stable and this result has also been confirmed in previous literature. (*J. Am. Chem. Soc.* **2015**, *137*, 11550; *Nature* **2013**, *501*, 399; *Nat. Commun.* **2013**, *4*, 2422)

As to their stability or instability, several previous studies on thiolate capped Ag_{44} nanoclusters have reported that Ag_{44} is prone to oxidation at room temperature, with oxidation occurring in a matter of hours (*Angew. Chem.*, **2009**, *121*, 6035; *Nanoscale*, **2012**, *4*, 4269; *J. Am. Chem. Soc.*, **2012**, *134*, 11856). This was also found in our experiment. Based on the results of our experiments, we speculate that Ag_{17} has similar properties and susceptibility to oxidation. On the other hand, Ag_{44} shows higher stability when capped by particular special thiolate ligands, such as 5-mercapto-2-nitrobenzoic acid (*J. Mater. Chem. A*, **2013**, *1*, 10148). This may help to explain the 'ultrastable' property of Ag_{44} reported in *Nature* **2013**, *501*, 399, where the nanoclusters were capped by 4-mercaptobenzoic acid.

Temperature and solvent also influence the stability of Ag_{44} nanoclusters. If we keep our final product in a freezer at -18 ^{o}C instead of room temperature, the final products remain stable for several days. If we dilute the reaction mixture with extra acetonitrile, the etching process proceeds to complete in 10 min at room temperature.

Supplementary References

1. Desireddy, A. *et al.* Ultrastable silver nanoparticles. *Nature* **501**, 399-402 (2013).
2. Harkness, K. M. *et al.* $Ag_{44}(SR)_{30}^{4-}$: a silver-thiolate superatom complex. *Nanoscale* **4**, 4269-4274 (2012).
3. Wickramasinghe, S. *et al.* $M_3Ag_{17}(SPh)_{12}$ Nanoparticles and Their Structure Prediction. *J. Am. Chem. Soc.* **137**, 11550-11553 (2015).
4. Tracy, J. B. *et al.* Electrospray ionization mass spectrometry of uniform and mixed monolayer nanoparticles: $Au_{25}[S(CH_2)_2Ph]_{18}$ and $Au_{25}[S(CH_2)_2Ph]_{18-x}(SR)_x$. *J. Am. Chem. Soc.* **129**, 16209-16215 (2007).
5. Zhu, M. Z., Qian, H. F. & Jin, R. C. Thiolate-Protected Au_{20} Clusters with a Large Energy Gap of 2.1 eV. *J. Am. Chem. Soc.* **131**, 7220-7221 (2009).
6. Zeng, C. J. *et al.* Total Structure and Electronic Properties of the Gold Nanocrystal $Au_{36}(SR)_{24}$. *Angew. Chem. Int. Ed.* **51**, 13114-13118 (2012).
7. Li, G., Zeng, C. J. & Jin, R. C. Thermally Robust $Au_{99}(SPh)_{42}$ Nanoclusters for Chemoselective Hydrogenation of Nitrobenzaldehyde Derivatives in Water. *J. Am. Chem. Soc.* **136**, 3673-3679 (2014).
8. Joshi, C. P., Bootharaju, M. S., Alhilaly, M. J. & Bakr, O. M. $[Ag_{25}(SR)_{18}]^-$: The "Golden" Silver Nanoparticle. *J. Am. Chem. Soc.* **137**, 11578-11581 (2015).
